# Into the Moment: Does Mindfulness Affect Biological Pathways in Multiple Sclerosis?

**DOI:** 10.3389/fnbeh.2018.00103

**Published:** 2018-05-22

**Authors:** Barbara Willekens, Gaetano Perrotta, Patrick Cras, Nathalie Cools

**Affiliations:** ^1^Department of Neurology, Antwerp University Hospital, Antwerp, Belgium; ^2^Laboratory of Experimental Hematology, Faculty of Medicine and Health Sciences, Vaccine & Infectious Disease Institute, University of Antwerp, Antwerp, Belgium; ^3^Department of Neurology, ULB-Hôpital Erasme, Brussels, Belgium; ^4^Department of Neurology, Translational Neurosciences, Faculty of Medicine and Health Sciences, University of Antwerp, Antwerp, Belgium; ^5^Department of Neurology, Laboratory for Neurobiology, Born-Bunge Institute, University of Antwerp, Antwerp, Belgium

**Keywords:** multiple sclerosis, mindfulness, fatigue, depression, cognitive function, immune system, stress, MRI

## Abstract

Mindfulness was introduced in the Western world by Jon Kabat-Zinn in 1979. He defined it as “awareness that arises through paying attention, on purpose, in the present moment, non-judgmentally.” Since then, research on mindfulness-based interventions (MBIs) has increased exponentially both in health and disease, including in patients with neurodegenerative diseases such as dementia and Parkinson's disease. Research on the effect of mindfulness and multiple sclerosis (MS) only recently gained interest. Several studies completed since 2010 provided evidence that mindfulness improves quality of life (QoL), depression and fatigue in MS patients. In addition to patient-reported outcome measures, potential effects on cognitive function have been investigated only to a very limited extent. However, research on laboratory biomarkers and neuroimaging, capable to deliver proof-of-concept of this behavioral treatment in MS, is mainly lacking. In this perspective, we illustrate possible neurobiological mechanisms, including the tripartite interaction between the brain, the immune system and neuroendocrine regulation, through which this treatment might affect multiple sclerosis symptoms. We propose to (1) include immunological and/or neuroimaging biomarkers as standard outcome measures in future research dedicated to mindfulness and MS to help explain the clinical improvements seen in fatigue and depression; (2) to investigate effects on enhancing cognitive reserve and cognitive function; and (3) to investigate the effects of mindfulness on the disease course in MS.

## Introduction

### Multiple sclerosis

Multiple sclerosis (MS) is a chronic neuroinflammatory and neurodegenerative disease with an unpredictable course that affects more than 2.5 million people worldwide. It is the main cause of nontraumatic disability in young adults in many countries (Browne et al., 2014). The disease course can be relapsing or progressive (Lublin et al., 2014). Varying visible (mainly physical) and unvisible or hidden symptoms occur in MS. The hidden symptoms, such as fatigue, depression, psychological distress, and cognitive dysfunction pose a significant burden on the patient's quality of life (QoL) and workforce participation. There is a high and unmet need for treatments able to tackle these hidden symptoms (Lysandropoulos and Havrdova, 2015). These invisible symptoms are highly prevalent in MS patients. Cognitive dysfunction occurs in 40–70%, depression in 30–40% while fatigue can be present in up to 85–95% of patients (Feinstein et al., 2014; Rocca et al., 2015; Patejdl et al., 2016). Cognitive dysfunction can start early in the disease course, even at the stage of clinically isolated syndrome (CIS) (Rocca et al., 2015; Benedict et al., 2017). The most frequently affected domains are information processing speed and episodic memory (Van Schependom et al., 2015; Hämäläinen and Rosti-Otajarvi, 2016; Köhler et al., 2017). Although self-reported cognitive dysfunction in patients with MS is only moderately correlated with objective cognitive impairment, it has a negative impact on QoL and is often related to mood and fatigue (Rosti-Otajärvi et al., 2014; Strober et al., 2016). It is well known that depression has adverse effects on cognitive functioning, including working memory, executive functioning, and information processing speed (Feinstein et al., 2014).

### Neurobiology of hidden symptoms in MS

The proposed underlying biological mechanisms for these hidden symptoms show some common and overlapping features. Neuroinflammation in the peripheral circulation and the central nervous system (CNS) has a role in cognitive dysfunction (Berger, 2016), fatigue (Hanken et al., 2014; Patejdl et al., 2016), but also in anxiety and depression in MS (Feinstein et al., 2014; Patejdl et al., 2016; Rossi et al., 2017). Interleukin-2 (IL-2), tumor necrosis factor-alpha (TNF-α) and interleukin-1 beta (IL-1β) in the cerebrospinal fluid (CSF) have been shown to correlate with mood disorders (Rossi et al., 2017), while serum interleukin-6 (IL-6) and increases in interferon-gamma (IFN-γ) and TNF-α (using whole-blood-cytokine-stimulation) correlate with fatigue (Patejdl et al., 2016). Growth factors may also play a role in the neurobiology of the invisible symptoms. A decrease in brain derived neurotrophic factor (BDNF) in early MS patients has been demonstrated and was associated with cognitive impairment in one recent study (Prokopova et al., 2017). It is well known that BDNF is a biomarker for depression, although this correlation remains to be shown in MS patients, whose immune cells secrete lower than normal BDNF (Azoulay et al., 2008; Kishi et al., 2017). After physical training in MS patients, an increase in BDNF can be paralleled by improved fatigue (Ozkul et al., 2018). Therapeutic strategies to decrease neuroinflammation and to increase BDNF might thus be helpful in alleviating hidden symptoms.

To date, serum biomarkers to monitor disease activity in MS don't exist, although neurofilament-light levels may hold promise in this regard (Novakova et al., 2017). Brain Magnetic Resonance Imaging (MRI) is currently regarded as a golden standard in MS to follow subclinical disease activity, consisting of gadolinium enhancing T1 lesions and new or enlarging T2 hyperintense lesions. MRI measurements of whole brain volume, and more specifically gray matter volume correlate well with disease progression, disability, and cognitive dysfunction (Rocca et al., 2015; Wattjes et al., 2015). On the other hand, MRI T2 lesion load is only weakly correlated with hidden symptoms (Feinstein et al., 2014; Rocca et al., 2015; Patejdl et al., 2016). MRI abnormalities may already occur before cognitive symptoms are noticeable (Roosendaal et al., 2010; Rocca et al., 2015). Hippocampal atrophy in MS patients has been correlated with both depression and cognitive dysfunction. This has been linked to increased cortisol levels (Feinstein et al., 2014). Both hippocampal and deep gray matter nuclei atrophy are predictors of cognitive impairment in MS and these measurements might be useful as biomarkers in treatment studies (Damjanovic et al., 2017; Köhler et al., 2017). While the correlation between certain structural MRI measures and cognition and depression shows common features, this is less clear for fatigue (Patejdl et al., 2016). MRI in fatigued MS patients correlates better with lesions and atrophy in prefrontal, thalamic and temporal structures (Patejdl et al., 2016). A decrease in the cortical thickness in the right middle temporal pole has been described in MS patients who are both fatigued and depressed (Hanken et al., 2016). To study brain plasticity, functional MRI (fMRI) is of interest (Enzinger et al., 2016). fMRI has led to the hypothesis that fatigue is a consequence of increased brain activation due to cortical reorganization, loss of connectivity and delayed processing, with important roles for the thalamus and frontoparietal cortex (Patejdl et al., 2016). Decreased connectivity in the anterior regions of the brain also plays a role in cognitive dysfunction and depression (Feinstein et al., 2014; Rocca et al., 2015).

To summarize, a proinflammatory cytokine profile and lower than normal BDNF levels seem to play a role in MS related fatigue, mood disorders, and cognitive function. Hippocampal and deep gray matter atrophy are correlated with cognitive dysfunction and depression, while fatigue is related to structural abnormalities in frontal, temporal but also deep gray matter regions.

### Treatment of hidden symptoms

The current treatment approach of cognitive dysfunction is to try to prevent cognitive impairment by using immunomodulatory drugs early in the disease course and when cognitive symptoms and/or impairment are present to resort to cognitive rehabilitation (Miller et al., 2017). Interestingly, perceived cognitive deficits can also improve after a neuropsychological rehabilitation program (Rosti-Otajärvi et al., 2013). Another preventive strategy is based on enhancing brain and cognitive reserve via a so-called brain-healthy lifestyle, including physical exercise (Crescentini et al., 2014; Sumowski et al., 2014; Sumowski, 2015; Sandroff et al., 2016; Kobelt and Giovannoni, 2017). Several studies have shown evidence for some beneficial effects of physical training on cognitive function, MRI measurements, neurotrophins, and/or immune markers in MS patients (Bansi et al., 2013; Leavitt et al., 2014; Kierkegaard et al., 2016; Wens et al., 2016; Feys et al., 2017; Kjølhede et al., 2017; Sandroff et al., 2017; Zimmer et al., 2017). A recent Cochrane review concluded that there is some evidence in favor of effectiveness of memory rehabilitation on memory function and on QoL. (Prosperini et al., 2015; das Nair et al., 2016; Hämäläinen and Rosti-Otajarvi, 2016). Non-pharmaceutical interventions to improve MS-related fatigue such as fatigue management and enhancing physical activity may be equally effective as pharmaceutical treatment such as amantadine (Brenner and Piehl, 2016; Tur, 2016). The treatment of depression in MS generally follows the guidelines for the general population, using pharmacological treatment with SSRI's (selective serotonin reuptake inhibitors) or CBT (cognitive behavioral therapy) (Brenner and Piehl, 2016). Despite these therapeutic approaches, hidden symptoms remain difficult to treat in clinical practice.

In conclusion, there are important relationships between subjective and objective cognitive dysfunction, depression, fatigue and QoL in MS and some of these symptoms show correlation with neuroimaging markers or immunological markers. Furthermore, there remains an unmet need for hypothesis-driven effective interventions that target these interrelated MS symptoms altogether and that have the ability to enhance brain and cognitive reserve.

## Mindfulness

Mindfulness was introduced in the Western world in 1979 by Jon Kabat-Zinn who defined it as “awareness that arises through paying attention, on purpose, in the present moment, non-judgmentally” (Kabat-Zinn, 1982). This meditation technique is based on traditional Buddhist meditation practices. The main goal is to enhance self-regulation via amplification of attentional control, improving emotional regulation and changing self-awareness (Tang et al., 2015). While the classic program, as developed by Jon Kabat-Zinn, finds its roots in mindfulness meditation and is named Mindfulness-Based Stress Reduction or MBSR, adapted programs are referred to as Mindfulness-Based Interventions or MBIs (Shonin et al., 2013) or Mindfulness Based Programs or MBPs (Crane et al., 2017). Since its introduction (Kabat-Zinn, 1982), numerous studies have been conducted on the immunological and neuroimaging effects of mindfulness in both health and disease, albeit not in MS. However, the quality of these studies has been highly variable due to inherent limitations of the intervention and control conditions (prone to bias due to self-report measures, short-term studies, small sample size, lacking active control groups, heterogeneity) (Goldberg et al., 2017; Kabat-Zinn, 2017).

As in MS, fatigue, cognitive symptoms and depression are often interrelated, mindfulness might be a way of dealing with all these problems at the same time (Senders et al., 2014). Here, we will review the beneficial effects of mindfulness that have been demonstrated in MS, describe effects of mindfulness on the immune system and MRI, explain the relationship between hidden symptoms in MS, immunology and MRI, elaborate on potential effects of mindfulness on cognitive function and disease course in MS, hypothesize on underlying neurobiological mechanisms, and finally suggest avenues for further research including laboratory biomarkers and neuroimaging.

### Clinical effects

To date, the feasibility of MBIs as an intervention for MS patients has been demonstrated in several studies (see Table 1). The studies comprised different types of MS patients, and incorporated a variety of MBIs, including even telemedicine, Skype, and/or online training (Grossman et al., 2010; Senders et al., 2014; Bogosian et al., 2015; Kolahkaj and Zargar, 2015; Frontario et al., 2016; Nejati et al., 2016; Blankespoor et al., 2017; Gilbertson and Klatt, 2017; Hoogerwerf et al., 2017; Simpson et al., 2017). A MBI has been shown to positively influence QoL, depression, anxiety and fatigue in MS patients in an RCT (intervention vs. waiting list control group) with a sample size of 150 patients and rigorous study design (pre- and post-intervention and 6 months follow-up) (Grossman et al., 2010). These beneficial effects have been confirmed in other RCTs that used an active control group (psychoeducational group) (Carletto et al., 2017; Cavalera et al., 2018). Moreover, sleep problems and illness perception improved to a greater extent in the intervention group than in the control group (Carletto et al., 2017; Cavalera et al., 2018). While the beneficial effects lasted for 6 months after conventional MBI (Carletto et al., 2017), the effects could not be maintained for 6 months with online mindfulness training (Cavalera et al., 2018).

**Table 1:** Mindfulness and MS in clinical studies.

**References**	**Study design**	**Sample size**	**Key inclusion criteria**	**Intervention**	**Control**	**Post-intervention follow-up**	**Primary outcome**	**Secondary outcome**	**Results**
Cavalera et al., 2018	RCT	139	RRMS and SPMS	8-week online course via Skype	Psychoeducational group	PI, 6 M	QoL	Anxiety and depression, sleep, fatigue	+ QoL, anxiety, depression, sleep at PI (– at 6 M)
Carletto et al., 2017	RCT	90	MS, depressive symptoms	Body-affective mindfulness	Psychoeducational group	PI, 6 M	Depression	Fatigue, perceived stress, illness perception	+ Depression, perceived stress, illness perception, QoL – Fatigue
Simpson et al., 2017	RCT	50	MS	MBSR	Waiting list	PI, 3 M	Feasibility, perceived stress, QoL	QoL, self-compassion, common MS symptoms	+ Perceived stress, anxiety, depression, self-compassion, positive affect at PI + Mindfulness, positive affect, self-compassion, anxiety, prospective memory at 3 M Feasible
Hoogerwerf et al., 2017	Non-randomized controlled	59	RRMS and SPMS, severe fatigue	MBCT	Patient is his/her own control	PI, 3 M	Fatigue	Anxiety and depression, coping, sleep, mindfulness	Feasible + Fatigue, anxiety, depression
Blankespoor et al., 2017	Open-label, pilot	25	MS	MBSR	No control	PI	Self-report and neuropsychological testing		+ Visual spatial processing, depressive symptoms, QoL, fatigue, mindfulness, self-compassion
Gilbertson and Klatt, 2017	Open-label, feasibility	20	MS	Mindfulness in motion	No control	PI	Feasibility	Fatigue, depression, anxiety, QoL	Feasible+ depression, anxiety, fatigue
Nejati et al., 2016	Controlled trial	24	MS	MBSR	Usual care?	PI	QoL, fatigue severity		+ QoL and fatigue severity
Frontario et al., 2016	Pilot RCT	30	MS	MBI based on MBSR, teleconference	One-time introduction to MBI	PI	SDMT and PASAT	Depression, fatigue	+ SDMT, PASAT, depression, and fatigue
Kolahkaj and Zargar, 2015	RCT	48	MS, females only	MBSR	Usual care	PI, 1 M	Anxiety, depression, stress		+ Anxiety, depression, stress
Bogosian et al., 2015	Pilot RCT	40	SPMS and PPMS	Mindfulness based on MBCT via Skype	Waiting list	PI, 3 M	Distress	Depression and anxiety, MS impact, pain, fatigue, QoL	+ Pain (only at 3 M), anxiety, depression, MS impact psychological
Grossman et al., 2010	RCT	150	RRMS and SPMS	MBI based on MBSR	Waiting list	PI, 6 M	QoL, depression, fatigue	Anxiety, perceived personal goal attainment, self-reported homework	+ QoL, well-being

*Overview of clinical studies investigating feasibility and/or efficacy of MBIs in MS since 2010. Study design, sample size, intervention, and control condition, follow-up, primary, and secondary outcomes and results are presented. RCT, randomized controlled trial; MS, all types of multiple sclerosis; RRMS, relapsing remitting MS; SPMS, secondary progressive MS; PPMS, primary progressive MS; MBSR, mindfulness based stress reduction; MBCT, mindfulness based cognitive therapy; MBI, mindfulness based intervention; PI, post-intervention; 1 M, 1 month; 3 M, 3 months; 6 M, 6 months; +, positive effect; –, no effect*.

There is some evidence to support positive effects of MBIs on cognition, including selective and executive attention, working memory, executive functions and cognitive flexibility, in healthy subjects (extensively reviewed by Chiesa et al., 2011). The effect of MBIs on cognition in MS patients has not been studied extensively, albeit its effect was explored in a number of pilot studies with variable results (Frontario et al., 2016; Blankespoor et al., 2017; Simpson et al., 2017). The first assessed the effects of a telemedicine mindfulness intervention in patients with MS in a randomized-controlled way and found that the Symbol Digit Modalities Test (SDMT) improved more in MS patients who underwent the intervention as compared to the control group who only had a one-time instruction (Frontario et al., 2016). In another pilot RCT, self-reported prospective memory had improved at 3 months post-intervention. However, the authors did not include objective cognitive measurements in this study (Simpson et al., 2017). More recently, a pilot study explored the effects of mindfulness on psychological functioning, QoL and cognitive function in a convenience sample of Dutch MS patients. In agreement with previous findings, significant improvements were found in depressive symptoms, QoL and fatigue. Moreover, improvement on a visual spatial processing test was reported after the intervention (Blankespoor et al., 2017). Also, Grossman et al. (2010) studied cognitive function, although this was not a primary nor key secondary outcome. The treating neurologist performed cognitive testing with the Multiple Sclerosis Inventory of Cognition (MUSIC), but results were not reported as there were no differences in these measures pre- and post-intervention nor at 6 months follow-up (*personal communication, P. Grossman*).

Although a more harmonized way of patient monitoring should be strived for, the above-mentioned observations underscore that MBIs should be considered as a valuable behavioral treatment option for MS patients capable to improve their mental well-being.

### Biological outcome measures

While a plethora of biological outcome measures are influenced by physical interventions in MS, including TNF-α, BDNF, IL-6, matrix metalloproteinases (MMP), soluble receptor of IL-6 (sIL-6R), nerve growth factor (NGF), and/or brain MRI measures (functional, gray matter) (Bansi et al., 2013; Leavitt et al., 2014; Deckx et al., 2015, 2016; Kierkegaard et al., 2016; Wens et al., 2016; Feys et al., 2017; Sandroff et al., 2017; Zimmer et al., 2017), research on the effects of mindfulness on biomarkers in MS remains elusive. Nonetheless, findings of MBIs in other study subjects, which will be outlined below, underscore the need to take into account soluble factors in future studies in MS.

#### Mindfulness and immunology

To date, there is only limited evidence that mindfulness meditation modulates the immune system. Mostly reported is a reduction in proinflammatory cytokine levels. Indeed, in a review of 20 RCTs of which 50% included an active control group and 50% a waiting list control group, a decrease in IL-6, TNF-α, and CRP as well as an increase in IL-8, IL-10, and IFN-γ was noted (Black and Slavich, 2016; Sanada et al., 2017). While in healthy subjects, MBIs had no effect on cytokine levels, except for an increase of insulin-like growth factor 1 (IGF-1) (Sanada et al., 2017), the effects were more pronounced in cancer patients, albeit with contradictory results including changes in the concentration of IFN-γ and IL-4, a decrease in TNF-α and IL-10, and no differences in the level of IL-6 (Sanada et al., 2017). One of the issues is that current markers have been measured in the peripheral blood rather than in the CNS and as such don't truly reflect the underlying biological process.

Furthermore, MBIs might have an impact on the expression of genes that are induced by stress. Several studies consistently showed a downregulation of the NFkB pathway, while in chronic stress this pathway is upregulated, leading to increased inflammation (Creswell et al., 2012; Kaliman et al., 2014; Buric et al., 2017). Hence, MBIs may lead to a reduction in NFkB-mediated inflammation. Interestingly, this pathway is also implicated in MS pathology and one of the current DMTs, dimethyl fumarate, modulates this pathway (Mc Guire et al., 2013; Miljković et al., 2015).

While one might expect a reduction in proinflammatory cytokines in serum and/or CSF as well as changes in gene expression of stress-induced genes in MS patients based on these data, this hypothesis remains to be investigated.

#### Mindfulness and MRI

A meta-analysis by Fox et al. (2014) reviewed 21 neuroimaging studies including approximately 300 meditation practitioners and found 8 brain regions that were most consistently changed in meditators. These were brain areas related to meta-awareness (frontopolar cortex), body awareness (sensory cortex and insula), memory (hippocampus), self and emotion regulation (anterior and mid cingulate; orbitofrontal cortex), and intra- and interhemispheric communication (superior longitudinal fasciculus; corpus callosum), all functions that are supposed to be enhanced through meditation. The global effect size was termed as medium (Cohen's *d* = 0.46; *r* = 0.19) (Fox et al., 2014). A more recent review (Gotink et al., 2016) on brain changes induced by mindfulness included 30 studies, concerning healthy subjects and patients. In both healthy as well as anxious and stressed participants, the activity, connectivity and volume of the prefrontal cortex, the cingulate cortex, the insula, and the hippocampus were increased. Specific changes in the amygdala included a decrease in functional activity, improvement in functional connectivity with the prefrontal cortex, and earlier deactivation after exposure to emotional stimuli. These results show similarities with the brain changes seen in meditation practitioners.

However, due to methodological limitations of the included studies and a concern for publication bias (Coronado-Montoya et al., 2016), more research is needed to confirm these findings. Of interest, brain regions that are influenced by MBIs are involved in hidden MS symptoms, as previously described. Adding structural and/or functional neuroimaging in research on the effects of MBIs in MS may improve the knowledge in this domain.

## How mindfulness might affect biological pathways and hidden symptoms in MS

To date, brain MRI nor biological samples have been included as outcome measures in studies on MBIs and MS to explain underlying neurobiological mechanisms of the positive outcomes (Grossman et al., 2010). Cognitive outcomes remain underexplored or underreported. However, some positive effects of MBIs on cognitive measures in Alzheimer's disease and hippocampal neuroplasticity in healthy people and Parkinson's disease have been observed in small studies (Pickut et al., 2013; Larouche et al., 2015; Quintana-Hernández et al., 2016). As described before, MBIs may induce changes in the brain that are of importance to emotional regulation and that might be relevant to enhance cognitive reserve or improve cognitive function in MS patients. To date, there is some preliminary evidence for a neuroprotective role from MBIs, potentially regulated by decreasing glucocorticoid levels and increasing BDNF (Tang et al., 2015). Thus, MBIs hold the potential to improve cognition in a diseased brain via structural brain changes that can be demonstrated directly post-intervention, as has been shown in a study on effects of a MBI in Parkinson's disease (Pickut et al., 2013).

Another way in which MBIs might improve the outcome for MS patients is by stress reduction, thereby influencing the disease course. Stress activates the hypothalamic-pituitary-adrenal axis (HPA-axis), to prepare the body for the “fight or flight” reaction. Although the role of stress in the risk of developing MS is debated, the literature on the association of relapses and stress is more consistent, pointing toward a higher risk of relapses after stressful life events (Artemiadis et al., 2011). Measuring stress however, is not easy and often relies on self-reporting. Biological outcome measures such as measuring autonomic nervous system response are another option (Briones-Buixassa et al., 2015). Stress reduction via a stress management program (SMT) can reduce brain inflammation in MS as measured by a decreased number of enhancing MRI lesions as compared to a control group in an RCT (Mohr et al., 2012). Moreover, MBIs are designed to reduce stress and have demonstrated an impact on several physiological markers of stress in different patient groups (Pascoe et al., 2017). Acute stress may disrupt blood-brain-barrier (BBB) permeability via corticotropine releasing hormone (CRH), neurotensin, and activate mast cells and microglia releasing pro-inflammatory cytokines and thereby attracting myelin-reactive T cells that cause MS relapses. Chronic stress, however, may lead to glucocorticoid resistance of immune cells (Gold and Heesen, 2006; Heesen et al., 2007a,b; Deckx et al., 2013; Karagkouni et al., 2013). In MS, hyperactivity of the HPA-axis has also been correlated with cognitive impairment (Heesen et al., 2002, 2010; Gold et al., 2005). A systematic review and meta-analysis studying physiological markers of stress following all types of meditation in different study populations showed that meditation reduced the serum levels of cortisol, C-reactive protein (CRP), triglycerides and TNF-α, as well as blood pressure and heart rate (Pascoe et al., 2017).

**Figure 1:**
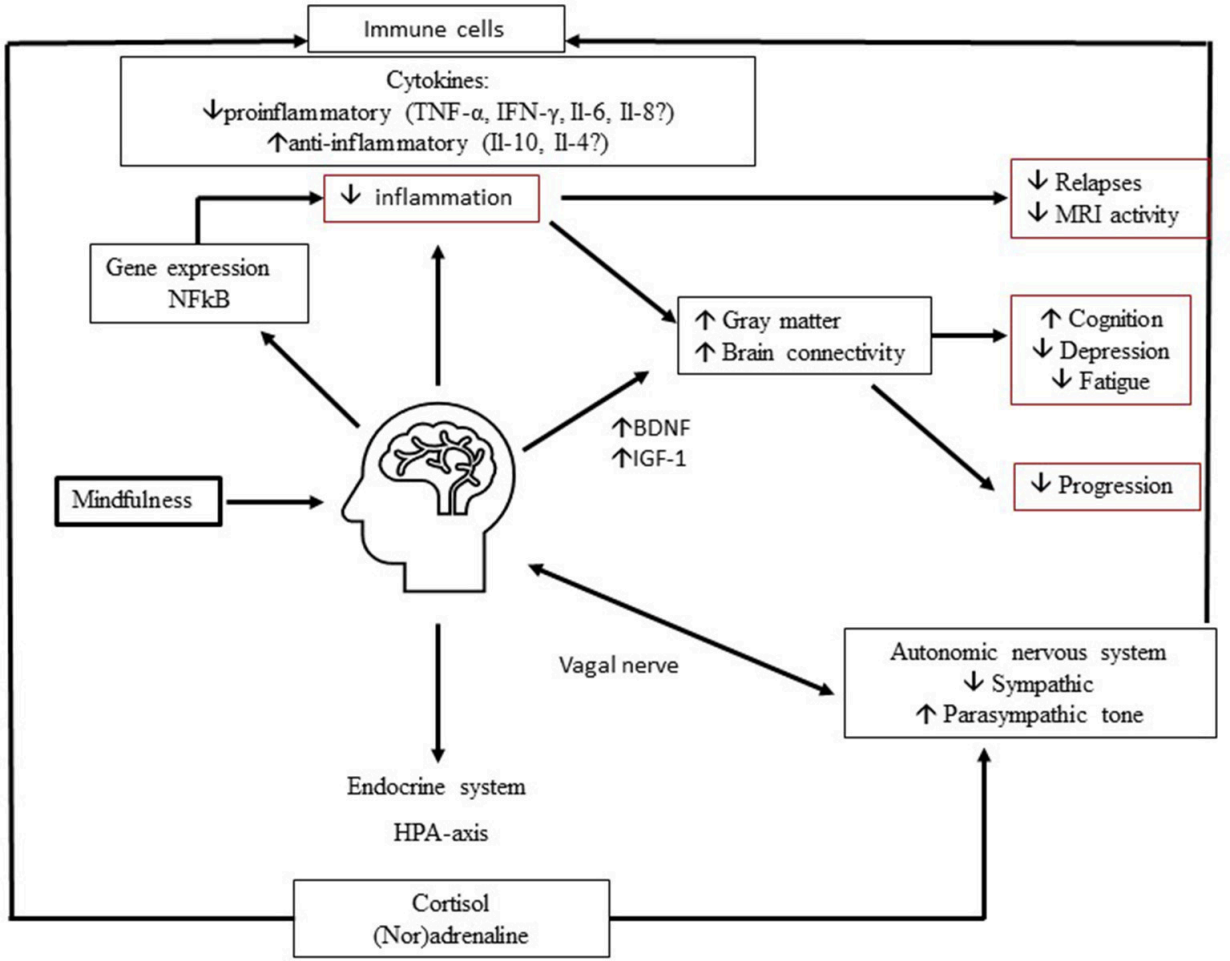
Proposed neurobiological mechanisms of mindfulness in MS. Mindfulness improves depression and fatigue and may improve cognitive function through complex multidirectional neurobiological mechanisms. Involvement of (1) endocrine system, (2) automatic nervous system, (3) growth factors, (4) gene expression may lead to decrease in inflammation and increase in Brain Derived Neurotrophic Factor (BDNF). In turn, this may lead to preserved and improved connectivity and gray matter volume. These mechanisms ultimately improve MS related invisible symptoms and possibly the disease course.

In Figure 1 we show a hypothetical framework to explain neurobiological effects of MBIs in MS. Depression and fatigue improve via a decrease in proinflammatory cytokines and an increase in anti-inflammatory cytokines, mediated via the endocrine system and autonomic nervous system. Also, changes in gene expression may lead to a decrease in inflammation and improvement in symptoms and maybe the disease course. Moreover, through increases in nerve growth factors like BDNF, brain connectivity and gray matter volume in certain brain regions may increase and lead to a decrease in depression, an improvement in fatigue and possibly improve cognitive function and slow down disease progression.

## Future directions and conclusion

In the past decade, the interest in neurobiological effects of behavioral interventions has rapidly increased. In MS, MBIs have demonstrated a positive impact on several hidden symptoms but also hold the potential to influence the disease course itself via various multidirectional brain- to -immune communications, including neuroendocrine, autonomic nervous system, immune and gene expression pathways leading to a decrease in inflammation and enhancing neuroplasticity (see Figure 1). MBIs may cause neuroplasticity in structures and functions of specific brain regions, important in attention, memory, emotional regulation, and self-awareness (Fox et al., 2014). In MS, some of these regions are implicated in pathophysiology of depression and fatigue, but also cognitive function. As therapeutic options to treat cognitive impairment in MS are limited, it is an interesting option to investigate effects of MBIs on enhancing cognitive reserve and cognitive function. Although current findings on research on MBIs and biomarkers must be interpreted with caution due to several limitations of the studies (prone to bias due to self-report measures, short-term studies, small sample size, lacking active control groups, heterogeneity), there are consistent effects seen in MRI and several soluble markers that require replication but also necessitate further in-depth research in the field of MS. While in research on exercise and MS, the use of biological outcome measures has taken an entry (Bansi et al., 2013; Leavitt et al., 2014; Kierkegaard et al., 2016; Wens et al., 2016; Feys et al., 2017; Sandroff et al., 2017; Zimmer et al., 2017), in studies on behavioral interventions and MS this is still a largely unexplored area. Therefore we propose to include immunological and/or neuroimaging biomarkers as standard outcome measures in future research dedicated to mindfulness and MS to help explain the clinical improvements seen in fatigue and depression. Effects of a MBI on cognitive function (subjective and objective) and biomarkers (MRI, cytokines, BDNF) are being investigated in the exploratory MIND-MS study (performed by the authors). This study involves 20 MS patients that undergo a MBI, in an open label study with 3 evaluation timepoints pre- and post-intervention and at 6 months, including cognitive outcomes, structural MRI and measurement of cytokines and BDNF. An RCT called REMIND-MS is currently ongoing and comparing the effects of cognitive rehabilitation with mindfulness vs. a control condition in the Netherlands, making use of MEG (magneto- encephalography) as secondary outcome measure (Nauta et al., 2017). It remains to be shown in MS patients, whether MBIs can have an impact on neuroinflammation and thus putatively on the disease course. Research on the effects of mindfulness on the disease course in MS, using conventional MRI measures and neurofilament-light levels should be done.

Mindfulness can only become an important adjuvant therapy to the current treatment modalities to improve outcomes in MS patients, when investigated in future well-designed clinical trials that follow the patients long-term, making use of not only clinical but also biological and imaging outcome measures, in doing so providing proof-of-concept of the neurobiological mode of action.

## Author contributions

BW concepted and drafted the manuscript; GP, PC, and NC critically revised the manuscript. All authors approved the submitted version of the manuscript.

### Conflict of interest statement

The authors declare that the research was conducted in the absence of any commercial or financial relationships that could be construed as a potential conflict of interest.
